# Research Trends in the Application of Yoga to Human Health: A Data Science Approach

**Published:** 2020-04

**Authors:** Chaitra Gururaja, D Rangaprakash, Gopikrishna Deshpande

**Affiliations:** 1 Department of Music, Auburn University Auburn, Alabama, USA; 2 Athinoula A. Martinos Center for Biomedical Imaging, Massachusetts General Hospital, Charlestown, Massachusetts, USA; 3 Department of Radiology, Harvard Medical School, Boston, Massachusetts, USA; 4 Division of Health Sciences and Technology, Harvard University and Massachusetts Institute of Technology, Cambridge, Massachusetts, USA; 5 AU MRI Research Center, Department of Electrical and Computer Engineering, Auburn University, Auburn, Alabama, USA; 6 Department of Psychology, Auburn University, Auburn, Alabama, USA; 7 Alabama Advanced Imaging Consortium, University of Alabama Birmingham, Alabama, USA; 8 Center for Health Ecology and Equity Research, Auburn University, Auburn, Alabama, USA; 9 Center for Neuroscience, Auburn University, Auburn, Alabama, USA; 10 School of Psychology, Capital Normal University, Beijing, China; 11 Key Laboratory for Learning and Cognition, Capital Normal University, Beijing, China; 12 Department of Psychiatry, National Institute of Mental Health and Neurosciences, Bangalore, India.

## Abstract

Yoga is an integrative mind-body system of wellbeing developed in India since at least three millennia. Yoga has gained considerable attention in recent decades, partly driven by recent research and evidence about its effectiveness. In this work, we extracted research trends on the effects of Yoga on human health from the US National Library of Medicine’s PubMed database (peer-reviewed journal papers). We found that Yoga research spans all organ systems and system-wide issues such as pain and cancer. Research on the nervous system far outpaces other systems, which is expected because of the effects of breathing and exercise on stress reduction, which has been a major application of Yoga. The next cluster of impact concerns the musculoskeletal system and pain (both related to the exercise [asana] aspects of Yoga), as well as cardiovascular/endocrine (also related to stress) and cancer. Stress and mental health, pain, diabetes, and cancer are health issues for which a permanent cure is not available in a majority of cases in modern medicine, although alleviating treatments are available. This has probably fueled interest in complementary approaches such as Yoga for these health issues. Research timeline shows that Yoga-related research largely expanded only after the 2000s. There was a specific uptick after 2004. Similar trends are seen if we look at just clinical trials or randomized control trials (RCTs) or systematic reviews. The percentage of trials (Clinical and RCT) among published literature is around 10–15 % This is comparable to other fields that gained traction around 2000s (e.g. non-invasive brain stimulation). Geographical distribution shows that 37% of all Yoga related research output originates in the USA, 19% from India, 13% from Europe and 31% from the rest of the world. Therefore, the interest is widespread and global. At least the uptick in Yoga-related research in the US post-2000s can be attributed to a substantial jump in funding between 1998 and 2005 from US National Institutes of Health’s National Center for Complementary and Integrative Health (NCCIH). We can only surmise that research in this field reached a critical mass in late-1990s, which infused more money into this field, generating more research and creating a positive feedback loop that has sustained the growth so far. We propose that in order to sustain or even accelerate future research in the area, rigor and reproducibility must be enhanced in addition to performing more RCT and clinical trials (increasing % of trials to 20–25% from 10–15%). The fruits of research in the field has to reach the common man in terms of evidence-based solutions to health issues. Without this, accelerated funding in democracies such as India and the USA will not be realizable.

## I. INTRODUCTION

Yoga is an integrative mind-body system of wellbeing invented in India at least three millennia ago [1]. This system has been preserved and advanced over time. In recent decades, Yoga has gained considerable attention outside India, especially in the west. This upsurge has been partly driven by recent research and evidence about the effectiveness of Yoga and its benefits to human health. For instance, Yoga has been found beneficial to reduce stress [2], enhance concentration [3], alleviate symptoms of asthma [4], provide palliative treatment to those with cancer [5], handle psychiatric disorders [6] [7], and more [8] [9]. In addition to globalization of Yoga through the efforts of Yoga experts and proponents (such as Yoga gurus) [10], we believe that scientific evidence has had a considerable contribution to bringing the benefits of Yoga to almost every corner of the world. Scientific evidence has considerably advanced our mechanistic understanding of the effects of Yoga on various facets of our body functioning. There is a need to understand the chronology of progression of Yoga research, as well as which physiological mechanisms and diseases have been most studied under Yoga. This will likely identify factors that have driven scientific research on Yoga and thus provide insights about how to sustain gains made so far and accelerate future research in the field. This study presents a numerical survey of the number of peer-reviewed journal papers published on Yoga in the past 70+ years. Specific emphasis is laid on the number of papers published since 2000, since when research interest in Yoga has been exponentially increasing. We also present how research has progressed in studying the effects of Yoga on different organ systems. It is notable that we only present trends in the number of journal publications and not the contents of those papers.

## II. METHODS

We confined our search to the U.S. National Library of Medicine’s PubMed database (https://pubmed.ncbi.nlm.nih.gov/). PubMed indexes papers from verified journals and publishers that adhere to minimum peer-review standards and is the largest database for biomedical research. Hence, PubMed was chosen, and we confined the search to only peer-reviewed journal publications. We extracted research trends on the effects of Yoga on human health from PubMed, which were available on the left panel under “*results by year*”, after searching the database with a search string.

We first explored all Yoga-related journal papers published within various organ systems and system wide domains. The following systems were considered: nervous, digestive, cardiovascular, immune, musculoskeletal, respiratory, urinary, reproductive, skin/integumentary and endocrine. Additionally, cancer and pain were considered as system-wide domains. PubMed shows papers with search terms present anywhere within a paper, in accordance with the logical search string used. The search terms used in PubMed were as follows (not case sensitive):

- Nervous: *(Yoga) AND ((brain) OR (nervous) OR (psychiatric) OR (neurological) OR (neuroscience) OR (psychology) OR (mental))*
- Digestive: *(Yoga) AND ((digestive) OR (stomach) OR (intestine) OR (liver))*
- Cardiovascular: *(Yoga) AND ((heart) OR (cardiovascular))*
- Immune: *(Yoga) AND ((immune) OR (immunity) OR (lymphatic))*
- Musculoskeletal: *(Yoga) AND ((Musculoskeletal) OR (bone) OR (muscle) OR (muscular) OR (balance))*
- Respiratory: *(Yoga) AND ((pulmonary) OR (lung) OR (respiratory))*
- Urinary: *(Yoga) AND ((urologic) OR (kidney) OR (renal) OR (urinary))*
- Reproductive: *(Yoga) AND ((reproductive) OR (ovary) OR (menopause) OR (testes) OR (sexual))*
- Skin: *(Yoga) AND (skin)*
- Endocrine: *(Yoga) AND ((diabetes) OR (endocrine) OR (metabolic))*
- Cancer: *(Yoga) AND (cancer)*
- Pain: *(Yoga) AND (pain)*

Next, we explored a subset of these papers that were only clinical trials. This was done by selecting the “*clinical trial*” and “*randomized controlled trial*” options under “article type” in the left panel in PubMed. After that, we explored review papers by selecting *“review*” and *“systematic review*” options. Furthermore, the geographical distribution of all published studies was examined as follows:

- India: *(Yoga) AND (India[Affiliation])*
- USA: *(Yoga) AND ((USA[Affiliation]) OR (America[Affiliation]) OR (United states[Affiliation]) OR (<state>[Affiliation]))*
  (Here, each of the 50 states was used in place of the *<state>* placeholder, which is not mentioned here for brevity).
- Europe: *(Yoga) AND ((Europe[Affiliation]) OR(<country>[Affiliation]))*
  (Here, each country in Europe was used in place of the *<country>* placeholder, which is not mentioned here for brevity).

In order to understand the reason for increased research throughput on Yoga (at least in part), we examined funding received towards Yoga-related research by the U.S. National Institutes of Health’s (NIH) National Center for Complementary and Integrative Health (NCCIH) (similar data was not available for other funding agencies outside USA). This was examined through published funding statistics from the NIH (https://www.nccih.nih.gov/about/budget/nccih-funding-appropriations-history).

In all cases, data representing the number of papers published per year was downloaded as a spreadsheet from the provided link under *“results by year”* on the left panel. These data were aggregated, plotted and interpreted.

## III. RESULTS

**Figure 1.**
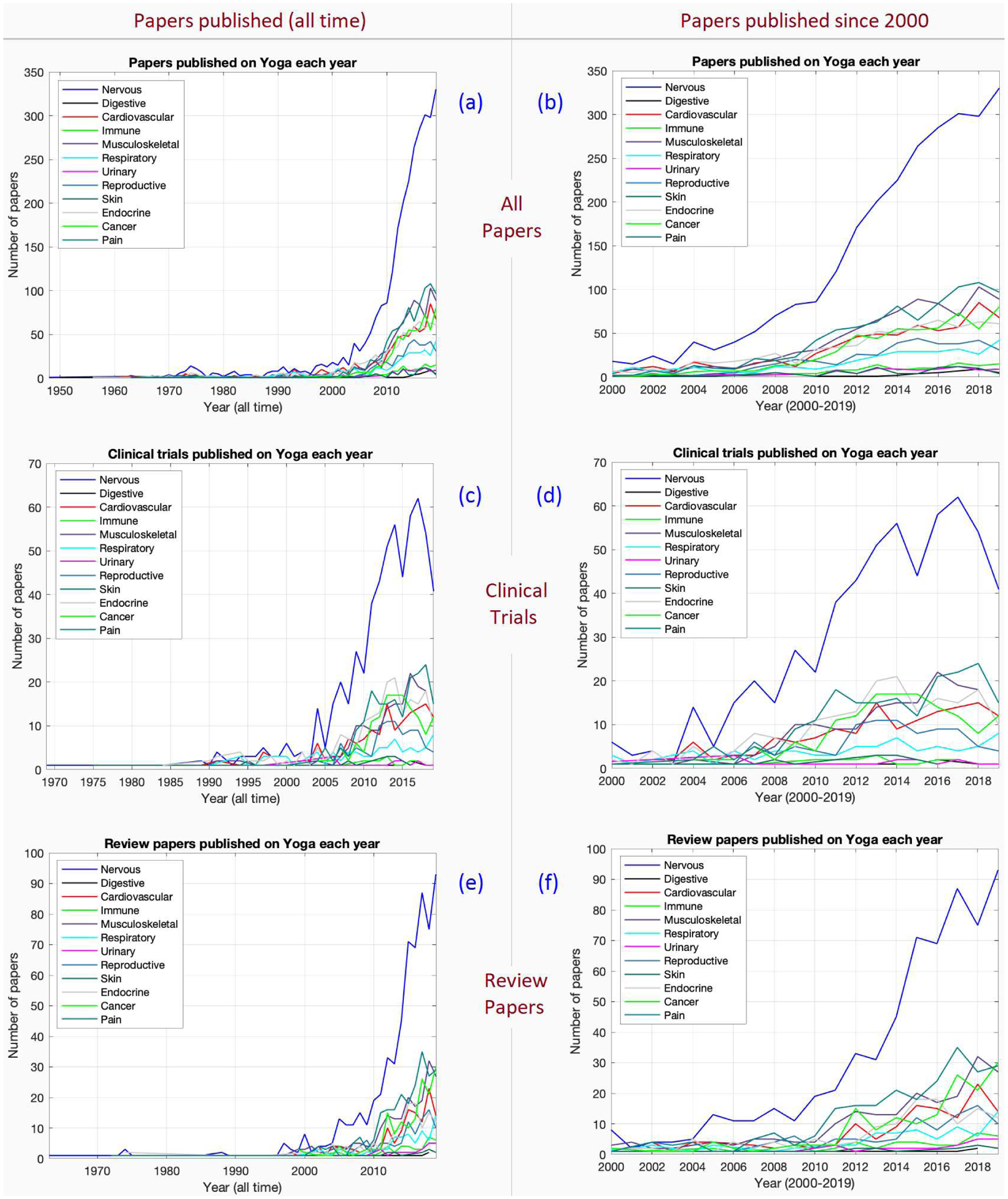
Research trends in Yoga; the graphs show papers published per year for various cases.

Results are presented for papers published across all time, as well as separately for papers published since the year 2000. We found that Yoga research spans all organ systems and system-wide issues such as pain and cancer (Figures 1a, 1b). Research on the nervous system far outpaces other systems. The next cluster of impact concerns the musculoskeletal system and pain (both related to the exercise [asana] aspects of Yoga), as well as cardiovascular/endocrine systems (also related to stress) and cancer.

Looking at the left column of figures (Figures 1a, 1c, 1e), the research timeline clearly shows that Yoga-related research largely expanded only after the 2000s. Hence, a closer look at years 2000–2019 has been presented in Figures 1b, 1d and 1e. Notably, there is a specific uptick after year 2004. Similar trends are seen with clinical trials or randomized control trials (RCTs) (Figures 1c, 1d) and systematic reviews (Figures 1e, 1f). The percentage of trials (Clinical and RCT) among published literature is around 10–15 %.

**Figure 2.**
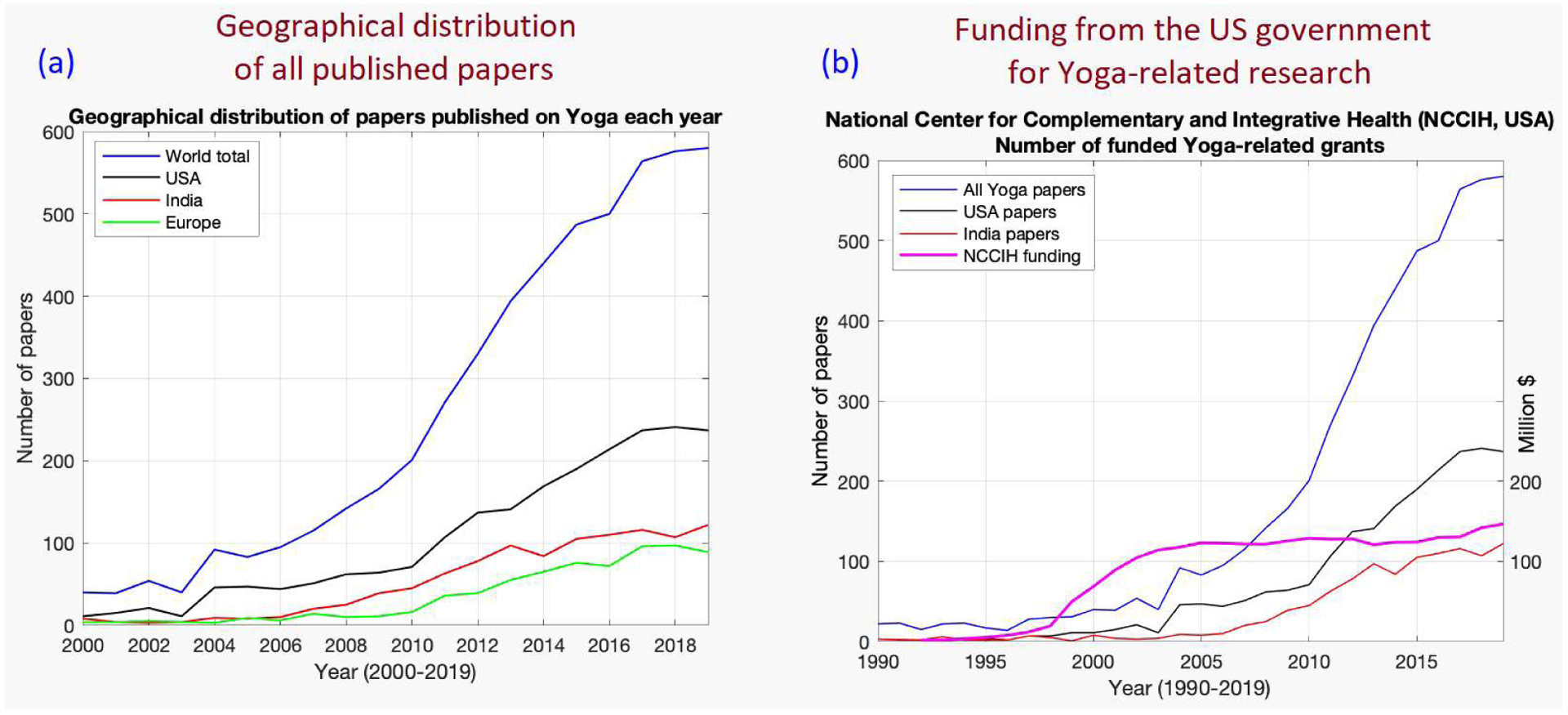
Geographical distribution of Yoga research and growth in funding from the US government for Yoga-related research.

Geographical distribution shows that 37% of all Yoga related research output originates in the USA, 19% from India, 13% from Europe and 31% from the rest of the world (Figure 2a). Therefore, the interest is widespread and global. NCCIH funding pattern (Figure 2b) shows substantial jump in funding between 1998 and 2005, which consolidated during years after 2005.

## IV. DISCUSSION

The aim of this study was to investigate trends in Yoga-related peer-reviewed research, which was achieved by examining PubMed for the number of published Yoga-related papers. Overall, we observed that Yoga-related research began in the 1950s (Figure 1a), but truly accelerated only after 2000 (Figure 1b). The stagnation prior to year 2000 is evident from the fact that 25 papers were published in 1973 and 31 papers were published in 1999 (literally no growth). However, while only 40 papers were published in the year 2003, nearly a 15-fold increase occurred during 2003–2019 with 580 papers published in 2019. Hence, the primary conclusion of our study is that Yoga-related research accelerated exponentially from 2004 onward.

Looking closer at Yoga-related research on different organ systems and domains, it is apparent that (Figure 1b) research on the nervous system far outpaces other systems. This is expected because of the effects of breathing and exercise on stress reduction, which has been a major application of Yoga. Other domains closely behind are endocrine (diabetes), cardiovascular, musculoskeletal and cancer. The musculoskeletal system (and pain) are related to the exercise (asana) aspects of Yoga; the cardiovascular/endocrine systems and cancer are related to the effects of Yoga on stress. Stress, mental health, pain, diabetes, and cancer are health issues for which a permanent cure is not available in a substantial portion of cases in modern medicine, although alleviating treatments are available. This has plausibly fueled interest in complementary approaches such as Yoga for these health issues.

Next, looking at trials (Clinical and RCT) (Figures 1c, 1d), their percentage among all published literature is around 10–15% (compare Figure 1d against Figure 1b). This is comparable to other fields that gained traction around 2000s [11] (e.g. non-invasive brain stimulation). Likewise, reviews account for about 30% of the literature (compare Figure 1f against Figure 1b), which again is comparable to other fields [11]. Thus, the progression and advancement of Yoga-related research has been similar to other emerging fields in the biomedical sciences.

The uptick in Yoga-related research in the US post-2000s can be attributed to a substantial jump in funding between 1998 and 2005 by NCCIH (Figure 2b). We can only surmise that research in this field reached a critical mass in late-1990s, which infused more money into this field, generating more research. This created a positive feedback loop that has sustained the growth so far. Comparable funding data is not publicly available in India (or elsewhere). We propose that in order to sustain or even accelerate future research in the area, rigor and reproducibility must be enhanced in addition to performing more RCT and clinical trials (increasing % of trials to 20–25% from 10–15%). The fruits of research in the field has to reach the common man in terms of evidence-based solutions to health issues. Without this, accelerated funding in democracies such as India and the USA will not be realizable.
